# New insights on the* Xylaria* species (*Ascomycota*, *Xylariales*) with bright-coloured exudates:* Xylaria aurantiorubroguttata* sp. nov. and revision of* X. haemorrhoidalis* and* X. anisopleura* type collections

**DOI:** 10.1186/s43008-024-00168-3

**Published:** 2024-11-21

**Authors:** Niccolò Forin, Alfredo Vizzini, Mario Amalfi, Samuele Voyron, Enrico Ercole, Simone Marcolini, Silvia Moschin, Barbara Baldan

**Affiliations:** 1 Department of Agronomy, Food, Natural Resources, Animals and Environment, DAFNAE, University of Padova, Viale Dell’Università 16, 35020 Legnaro, Italy; 2 Botanical Garden, University of Padova, Via Orto Botanico 15, 35123 Padua, Italy; 3 Department of Life Sciences and Systems Biology, University of Torino, Viale P.A. Mattioli 25, 10125 Turin, Italy; 4 Meise Botanic Garden, Nieuwelaan 38, 1860 Meise, Belgium; 5 Fédération Wallonie-Bruxelles, Service Général de L’Enseignement Supérieur Et de La Recherche Scientifique, 1080 Brussels, Belgium; 6 Via Murazzano 11, 10141 Turin, Italy; 7 Sant’Anna School of Advanced Studies, Institute of Crop Science, Via Luigi Alamanni 22, 56010 San Giuliano Terme, Italy; 8 Department of Biology, University of Padova, Via Ugo Bassi 58B, 35121 Padua, Italy

**Keywords:** *Sordariomycetes*, *Xylariaceae*, *Xylaria aurantiorubroguttata* new taxon, *Xylaria polymorpha* complex, Taxonomy

## Abstract

A new species of *Xylaria* is described based on morphological characters of both sexual and asexual morphs, and molecular data based on nuclear rDNA internal transcribed spacer, α-actin, β-tubulin and RNA polymerase subunit II sequences. *Xylaria aurantiorubroguttata* is characterized by the presence of both upright, cylindrical, long-stipitate and globose to subglobose, short-stipitate stromata, immature stromatal stages producing at first orange and then red drops, and ascospores with a slightly oblique, straight half spore-length germ slit. We provide also new morphological descriptions for *X. haemorrhoidalis* (holotype) and *X. anisopleura* (isosyntype), two *Xylaria* species belonging to *X. polymorpha* complex together with *X. aurantiorubroguttata*.

## INTRODUCTION

*Xylaria* Hill ex Schrank, typified with *X. hypoxylon* (L.) Grev., is the largest and representative genus of the family *Xylariaceae*, although the exact number of species within this genus is not yet clear due to the lack of a world monograph. The majority of *Xylaria* species are characterized by upright, cylindrical to clavate and multiperitheciate stromata; asci cylindrical, long-pedicellate, with eight spores uniseriate in the ascus and an amyloid apical apparatus; dark brown, ellipsoid-inequilateral ascospores with a germ slit; and a geniculosporium-like asexual morph (Daranagama et al. 2018; Konta et al. 2020). Nevertheless, some species have sessile, wider than high stromata (named penzigioid) or crowded aggregate stromata forming a crust on the substrate (e.g., *X. heliscus* (Mont.) J.D. Rogers and Y.M. Ju). They grow up on decayed wood, fallen leaves, petioles, fruits and seeds, soil or associated with living plants or termite nests (Hsieh et al. 2010, 2022; Ju et al. 2018; Ma et al. 2020; Wangsawat et al. 2021; Ju and Hsieh 2023).

Phylogenetically, the genus *Xylaria* results polyphyletic (Daranagama et al. 2018; Wendt et al. 2018; Pan et al. 2022); however, *Xylaria* species can be subdivided in three major clades defined as: “HY” clade represented by *X. hypoxylon*, “PO” clade represented by *X. polymorpha* (Pers.: Fr.) Grev. and “TE” clade (= subgenus *Pseudoxylaria*) that comprises only species associated with termite nests (Hsieh et al. 2010). Within the clades, different species aggregates have been recognized based on specific morphological characters. *X. polymorpha* aggregate includes species, such as *X. globosa* (Spreng.: Fr.) Mont. or *X. haemorrhoidalis* Berk and Broome, with finely cracked and wrinkled stromatal surface and ascospores 17–35 μm long, forming a monophyletic group in the “PO” clade (Hsieh et al. 2010). Some species of the *X. polymorpha* aggregate have a greenish asexual morph developing on immature stromata (Rogers 1985; Rogers and Callan 1986; Fournier et al. 2021).

In the present paper a new *Xylaria* species belonging morphologically to the *X. polymorpha* aggregate with immature stromata exuding orange-red drops is described. Its morphological and culture characteristics are investigated, and ITS, *ACT*, *TUB2* and *RPB2* sequences analyzed to confirm its placement in the *X. polymorpha* aggregate. In order to compare its morphology with closely related species, new macro- and micro- morphological observations of *X. haemorrhoidalis* and* X. anisopleura* (Mont.) Fr. were made based on type material.

## MATERIALS AND METHODS

### Sample collection and morphology

**Fig. 1:**
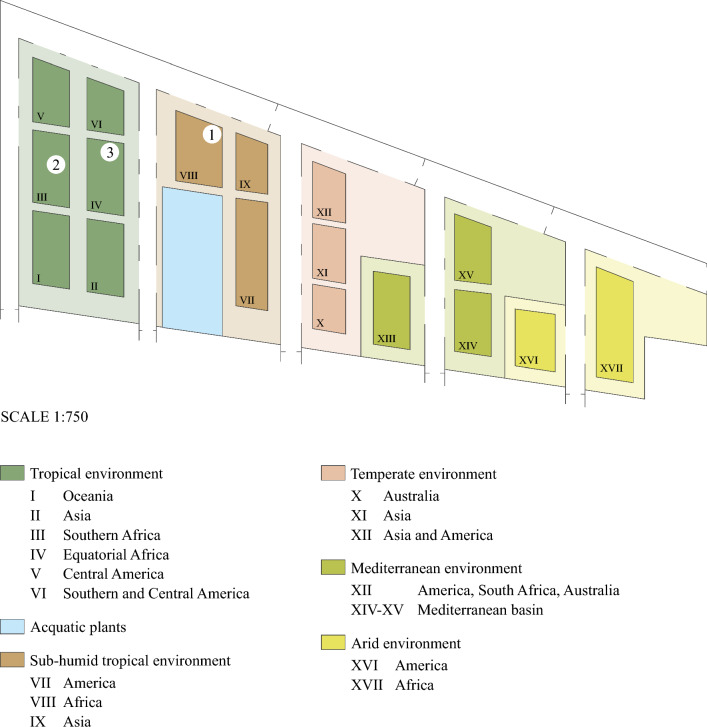
Plan of the Padova Botanical Garden’s greenhouses (scale 1:750). The different environments are indicated with different colours, and they are subdivided in geographical regions depending on the origin of plants. The locations of the three *Xylaria aurantiorubroguttata* specimens are reported: (1) PAD H0061559, holotype; (2) PAD H0061560, paratype; (3) PAD H0061561, paratype

Stromata were collected in three areas of Padova Botanical Garden’s tropical greenhouses from: a stump of *Delonix regia* (*Fabaceae*) in the sub-humid tropical environment (specimen 1 in Fig. 1); a cut trunk of *Phoenix reclinata* (*Arecaceae*) (specimen 2 in Fig. 1) and at the base of a bamboo rod used as support for *Strophanthus speciosus* (*Apocynaceae*) in the tropical environment (specimen 3 in Fig. 1).

The morphological characteristics of stromatal surface, perithecia and ascospores were observed under a stereomicroscope (Leica EZ4W) and environmental scanning electron microscope (ESEM). Sections of perithecia were obtained after the fixation and embedding of stromata following the protocol reported in D’Apice et al. (2021). Sections of 8–10 μm were cut on a Leica RM 2125 RTS microtome, deparaffinized, rehydrated and stained with 1% (w/v) toluidine blue. Slides were observed with an optical microscope (Leica DM500) with 400 × or 1000 × magnifications and photographed with a camera integrated in the microscope (Leica ICC50W).

Asci, ascospores, conidiogenous cells and conidia were observed under an optical microscope (Leica DM500) and photographed with its integrated camera (Leica ICC50W) after adding 3% lactic acid solution of Cotton Blue. The amyloid reaction of the ascus apical apparatus was tested pre-treating some perithecia with 10% potassium hydroxide (KOH) and then with Lugol’s solution. The measurements of micromorphological elements were taken using Fiji (Schindelin et al. 2012). Measures of ascospores and conidia are indicated as: (minimum–) average minus standard deviation–*average*–average plus standard deviation (–maximum) of length × (minimum–) average minus standard deviation–*average*–average plus standard deviation (–maximum) of width. In addition, spore quotient (Q; length/width ratio) = (minimum–) average minus standard deviation–*average*–average plus standard deviation (–maximum) is reported. Cultures were obtained by inoculating, under sterile condition, small pieces from the inner stromata tissue in 9 cm Petri dishes containing Malt Extract Agar culture media (MEA, 20 g/L malt extract, 20 g/L glucose, 1 g/L peptone from casein, 1 L distilled water) and streptomycin 0.015 g/L. The antibiotic was added to the sterilized culture medium when it had reached 50 °C in order not to compromise the functionality of the streptomycin. Petri dishes with the stromata were then incubated at 25 °C.

Dried specimens are stored at the Botanical Garden of Padova (Herbarium Code PAD), Italy; while living culture at the Mycotheca Universitatis Taurinensis (MUT) in the Botanical Garden of Torino (Herbarium Code TO), Italy.

### DNA extraction, PCR amplification and DNA sequencing

Genomic DNAs were extracted from fresh stromata of the new species using the CTAB protocol described in Forin et al. (2018). The internal transcribed spacer region (ITS) of nuclear rDNA was amplified with the primers ITS1F/ITS4 (White et al. 1990; Gardes and Bruns 1993), beta-tubulin gene (*TUB2*) with the primers T1/T22 (O’Donnell and Cigelnik 1997), alpha-actin gene with the primers ACT-512F/ACT-783R (Carbone and Kohn 1999), while the RNA polymerase II second largest subunit (*RPB2*) was amplified with the primers fRPB2-5F/fRPB2-7cR (Liu et al. 1999). Amplification reactions were performed in a total volume of 25 μL reaction mixture including: 5 μL of 5X Wonder Taq reaction buffer (EuroClone; 5 mM dNTPs, 15 mM MgCl_2_), 0.5 μL of bovine serum albumin (BSA, 10 mg/mL), 0.5 μL each of two primers (10 μM), 0.5 μL of Wonder Taq (5 U/μL), 2 μL of genomic DNA and water to reach the final volume. The PCR programs were as follows: 3 min at 95 °C for one cycle; 30 s at 95 °C, 40 s at 50–55 °C, 45 s at 72 °C for 35 cycles; 5 min at 72 °C for one cycle. The PCR products were enzymatically purified using ExoSAP-IT™ PCR Product Cleanup Reagent (Thermo Fisher) following the manufacturer’s protocol. The purified products were quantified with Qubit dsDNA HS Assay Kit (Thermo Fisher) and sequenced by Eurofins Genomics company (Vimodrone, Milan, IT). The new generated sequences were deposited in GenBank under the accession numbers reported in Table 1.

**Table 1:** Sequences and species used in the combined phylogenetic analysis, including specimen number, country of origin and GenBank accession numbers

Species	Strain/voucher	Country	GenBank accession numbers				References
			ITS	*ACT*	*TUB*	*RPB2*	
*Xylaria allantoidea*	HAST 94042903	Taiwan	GU324743	GQ452377	GQ502692	GQ848356	Hsieh et al. (2010)
*X. anisopleura*	isolate 938	Ecuador	KP133317	–	–	–	Thomas et al. (2016)
*X. anisopleura*	isolate 1074	Ecuador	KP133318	–	–	–	Thomas et al. (2016)
*X. apoda*	HAST 90080804	Taiwan	GU322437	GQ438751	GQ495930	GQ844823	Hsieh et al. (2010)
*X. atrosphaerica*	HAST 91111214	Taiwan	GU322459	GQ452363	GQ495953	GQ848342	Hsieh et al. (2010)
***X. aurantiorubroguttata***	**PAD H0061559**	**Italy**	**PQ456798**	**PQ464564**	**PQ464570**	**PQ464567**	**This study**
***X. aurantiorubroguttata***	**PAD H0061560**	**Italy**	**PQ456799**	**PQ464565**	**PQ464571**	**PQ464568**	**This study**
***X. aurantiorubroguttata***	**PAD H0061561**	**Italy**	**PQ456800**	**PQ464566**	**PQ464572**	**PQ464569**	**This study**
*X. badia*	HAST 95070101	Taiwan	GU322446	GQ449235	GQ495939	GQ844833	Hsieh et al. (2010)
*X. berteri*	JDR 256	USA	GU324750	GQ455442	GQ502698	GQ848363	Hsieh et al. (2010)
*X. berteri*	YMJ 90112623	Taiwan	GU324749	AY951874	AY951763	GQ848362	Hsieh et al. (2005, 2010)
*X. castorea*	PDD 600	New Zealand	PQ005705	GQ455447	GQ502703	GQ853018	Hsieh et al. (2010)
*X.* cf. *castorea*	HAST 91092303	Taiwan	GU324752	GQ455448	GQ502704	GQ853019	Hsieh et al. (2010)
*X. crozonensis*	HAST 398	France	GU324748	GQ455441	GQ502697	GQ848361	Hsieh et al. (2010)
*X. cubensis*	HAST 515	Martinique	GU373810	GQ455445	GQ502701	GQ848366	Hsieh et al. (2010)
*X. cubensis*	JDR 860	USA	GU991523	GQ455444	GQ502700	GQ848365	Hsieh et al. (2010)
*X. culleniae*	JDR 189	Thailand	GU322442	GQ438756	GQ495935	GQ844829	Hsieh et al. (2010)
*X. curta*	HAST 494	Martinique	GU322444	GQ449233	GQ495937	GQ844831	Hsieh et al. (2010)
*X. digitata*	HAST 919	Ukraine	GU322456	GQ449245	GQ495949	GQ848338	Hsieh et al. (2010)
*X. enterogena*	HAST 785	French Guiana	GU324736	GQ452370	GQ502685	GQ848349	Hsieh et al. (2010)
*X. feejeensis*	HAST 92092013	Taiwan	GU322454	GQ449243	GQ495947	GQ848336	Hsieh et al. (2010)
*X. frustulosa*	HAST 92092010	Taiwan	GU322451	GQ449240	GQ495944	GQ844838	Hsieh et al. (2010)
*X.* cf. *glebulosa*	HAST 431	Martinique	GU322462	GQ452366	GQ495956	GQ848345	Hsieh et al. (2010)
*X. globosa*	HAST 775	Guadeloupe	GU324735	GQ452369	GQ502684	GQ848348	Hsieh et al. (2010)
*X. globosa*	isolate 1095	Ecuador	KP133419	–	–	–	Thomas et al. (2016)
*X. globosa*	isolate 980	Ecuador	KP133425	–	–	–	Thomas et al. (2016)
*X. haemorrhoidalis*	HAST 89041207	Taiwan	GU322464	GQ452368	GQ502683	GQ848347	Hsieh et al. (2010)
*X. haemorrhoidalis*	FS108	–	MF770875	–	–	–	Direct submission
*X.* cf. *heliscus*	HAST 88113010	Taiwan	GU324742	GQ452376	GQ502691	GQ848355	Hsieh et al. (2010)
*X. ianthinovelutina*	HAST 553	Martinique	GU322441	GQ438755	GQ495934	GQ844828	Hsieh et al. (2010)
*X. intracolorata*	HAST 90080402	Taiwan	GU324741	GQ452375	GQ502690	GQ848354	Hsieh et al. (2010)
*X. laevis*	HAST 419	Martinique	GU324746	GQ455439	GQ502695	GQ848359	Hsieh et al. (2010)
*X. laevis*	HAST 95072910	Taiwan	GU324747	GQ455440	GQ502696	GQ848360	Hsieh et al. (2010)
*X. luteostromata* var. *macrospora*	HAST 508	Martinique	GU324739	GQ452373	GQ502688	GQ848352	Hsieh et al. (2010)
*X. montagnei*	HAST 495	Martinique	GU322455	GQ449244	GQ495948	GQ848337	Hsieh et al. (2010)
*X. muscula*	HAST 520	Guadeloupe	GU300087	GQ408909	GQ478222	GQ844800	Hsieh et al. (2010)
*X. ophiopoda*	HAST 93082805	Taiwan	GU322461	GQ452365	GQ495955	GQ848344	Hsieh et al. (2010)
*X. oxyacanthae*	JDR 859	USA	GU322434	GQ438748	GQ495927	GQ844820	Hsieh et al. (2010)
*X. palmicola*	PDD 604	New Zealand	GU322436	GQ438750	GQ495929	GQ844822	Hsieh et al. (2010)
*X. phyllocharis*	HAST 528	Guadeloupe	GU322445	GQ449234	GQ495938	GQ844832	Hsieh et al. (2010)
*X. plebeja*	HAST 91122401	Taiwan	GU324740	GQ452374	GQ502689	GQ848353	Hsieh et al. (2010)
*X. polymorpha*	JDR 1012	USA	GU322460	GQ452364	GQ495954	GQ848343	Hsieh et al. (2010)
*X. polymorpha*	TW07032019_02	USA	MN846336	MN917752	–	MN917814	Garcia-Aroca et al. (2021)
*X. regalis*	HAST 92072001	Taiwan	GU324744	GQ452378	GQ502693	GQ848357	Hsieh et al. (2010)
*X. schweinitzii*	HAST 92092023	Taiwan	GU322463	GQ452367	GQ495957	GQ848346	Hsieh et al. (2010)
*X. schweinitzii*	isolate 904	Ecuador	KP133472	–	–	–	Thomas et al. (2016)
*X. scruposa*	HAST 497	Martinique	GU322458	GQ452362	GQ495952	GQ848341	Hsieh et al. (2010)
*X. spinulosa*	GZUCC13016	China	–	KM236097	KM236099	KM236098	Li et al. (2017)
*X. telfairii*	HAST 421	Martinique	GU324737	GQ452371	GQ502686	GQ848350	Hsieh et al. (2010)
*X. telfairii*	HAST 90081901	Taiwan	GU324738	GQ452372	GQ502687	GQ848351	Hsieh et al. (2010)

The new sequences from this study are in bold

The DNA was also extracted from *X. haemorrhoidalis* and *X. anisopleura*. Due to the age of the specimens, an Illumina sequencing was applied to obtain ITS1 and/or ITS2 sequences following the protocol reported in Forin et al. (2020). Unfortunately, the sequencing failed in retrieving ITS information from these types.

### Phylogenetic analyses

**Fig. 2:**
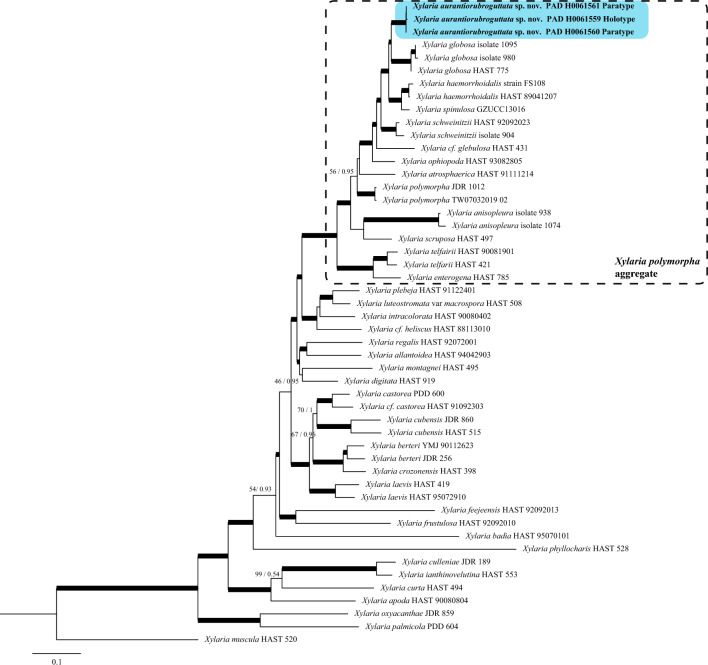
Phylogenetic tree of selected *Xylaria* species based on a combined ITS-*ACT-TUB2-RPB2*-introns dataset. Thickened branches represent bootstrap value (ML BS) and BPP value greater than 75%/0.95. For selected nodes parsimony bootstrap support value and Bayesian posterior probabilities are indicated respectively to the left and right of slashes. The new species X*. aurantiorubroguttata* is in bold

**Fig. 3:**
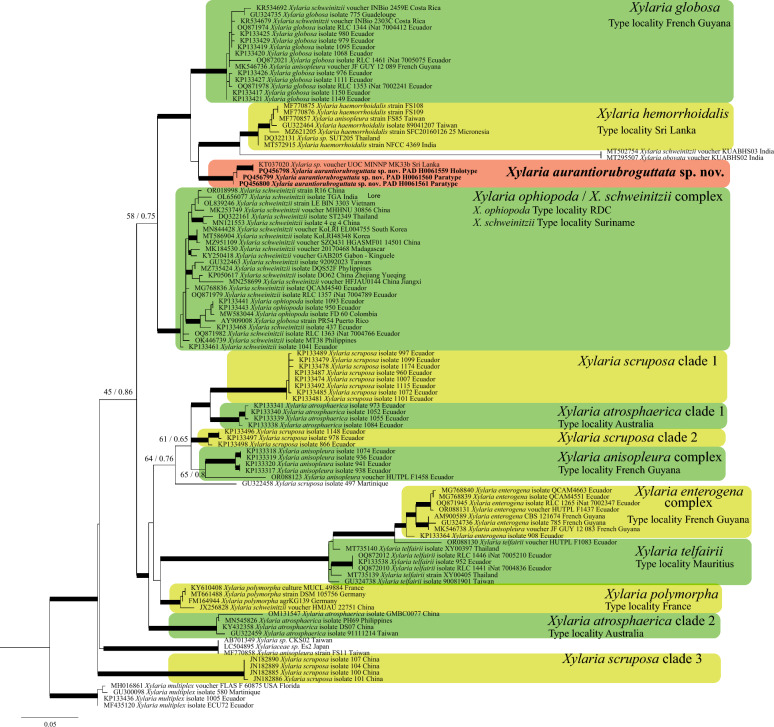
Phylogenetic tree based on ITS dataset and circumscribed to *Xylaria* species of the *X. polymorpha* aggregate. Thickened branches represent bootstrap value (ML BS) and BPP value greater than 75%/0.95. For selected nodes parsimony bootstrap support value and Bayesian posterior probabilities are indicated respectively to the left and right of slashes. The new species *X. aurantiorubroguttata* is in bold

The sequences obtained in this study were compared to those deposited in GenBank using the BLASTn algorithm. Based on the BLASTn results and the outcomes of phylogenetic studies focused on *Xylaria* (Hsieh et al. 2010; Li et al. 2017; Garcia-Aroca et al. 2021; Wangsawat et al. 2021; Pan et al. 2022), the sequences were retrieved from GenBank to perform a multi-gene phylogenetic analysis (Table 1, Fig. 2) limited to the PO clade (Hsieh et al. 2010) and an ITS-only phylogenetic inference comprising 108 sequences including the outgroup limited to the *X. polymorpha* aggregate (Fig. 3).

Nucleotide sequences were automatically aligned with the MUSCLE algorithm (Edgar 2004) with default settings, then manually adjusted as necessary with PhyDE® v0.9971 (Müller et al. 2010). Potentially ambiguously aligned segments were also detected using the Gblocks v0.91b program (Castresana 2000) with the following parameter settings: minimum number of sequences for a conserved position = 26 (minimum possible); minimum number of sequences for a flank position = 26 (minimum possible); maximum number of contiguous non-conserved positions = 5 bp, minimum block size = 5 bp and gaps allowed within selected blocks in half of the sequences. The assignment of codon positions was confirmed by translating nucleotide sequences into predicted amino acid sequences using MacClade 4.0 (Maddison and Maddison 2005) and then compared with the annotated *Xylaria* sequences available on GenBank.

Phylogenetic analyses were performed separately for each individual and concatenated loci using Bayesian inference (BI) as implemented in MrBayes v3.2 (Ronquist et al. 2012) and Maximum likelihood (ML) as implemented in RAxML 7.0.4 (Stamatakis 2006; Stamatakis et al. 2008).* Xylaria muscula* Lloyd was chosen as outgroup of the PO clade following Hsieh et al. (2010) for the combined dataset and* X. multiplex* (Kunze) Fr. as outgroup of the restricted dataset following the phylogenetic results of the combined dataset. Models of evolution for BI were estimated using the Akaike information criterion (AIC) as implemented in jModelTest v. 2.1.7 (Darriba et al. 2012). In order to facilitate the data partitioning by codon position, the introns of each coding region were excised and analysed altogether as a distinct partition. Therefore, the dataset was subdivided into 8 partitions: (ITS) (*ACT* codons 1 + 2) (*ACT* codon 3) (*TUB2* codons 1 + 2) (*TUB2* codon 3) (*RPB2* codons 1 + 2) (*RPB2* codon 3) (introns). The best-fit models for each partition were implemented as partition-specific models within partitioned mixed-model analyses of the combined dataset and all parameters were unlinked across partitions. The combined dataset Bayesian analyses were implemented with four independent runs, each with four simultaneous independent chains for 10 million generations, starting from random trees, and keeping one tree every 1000th generation. All trees sampled after convergence (ave. standard deviation of split frequencies < 0.01) and confirmed using Tracer v1.4 (Rambaut and Drummond 2007) were used to reconstruct a 50% majority-rule consensus tree (BC) and to calculate Bayesian posterior probabilities (BPP). BPP of each node was estimated based on the frequency at which the node was resolved among the sampled trees with the consensus option of 50% majority-rule (Simmons et al. 2004). A probability of 0.95 was considered significant. Maximum likelihood (ML) searches were conducted with RAxML involving 1000 replicates under the GTRGAMMAI model, with all model parameters estimated by the program. In addition, 1000 bootstrap (ML BS) replicates were run with the same GTRGAMMAI model. In order to force RaxML software to search for a separate evolution model for each dataset, we provided an additional alignment partition file to the software. Clades with ML BS values of 75% or greater were considered supported by the data. Nucleotide sequences are considered to be phylogenetically informative until they reach the substitution saturation, especially in coding sequences, saturation will be more pronounced in the rapidly evolving third codon position. At this point, it is no longer possible to deduce whether an observed similarity between a pair of sequences results from their common ancestry or whether this has occurred by chance (Jeffroy et al. 2006). To detect the possible bias from substitution saturation, we tested the first, second and the third codon position of the coding region studied as well as the non-coding loci by using Xia’s test (Xia et al. 2003; Xia and Lemey 2009), as implemented in DAMBE (Xia and Xie 2001). Because the critical index substitution saturation (Iss.c) is based on simulation results, there is a problem with more than 32 species. To circumvent this problem, DAMBE was used to randomly sample subsets of 4, 8, 16 and 32 OTUs multiple times and perform the test for each subset to see if substitution saturation exists for these subsets of sequences. In order to confirm the results of the Xia’s method we also plotted transitions and transversions at the first, second, and third codon positions against Tamura-Nei genetic distances with the aid of the DAMBE package, with an asymptotic relationship indicating the presence of saturation.

Before combining the data partitions, topological incongruence between the datasets was assessed using 1000 replicates of ML BS under the same models described above, on each locus separately. Paired trees were examined for conflicts only involving nodes with ML BS > 75% (Mason-Gamer and Kellogg 1996; Reeb et al. 2004; Lutzoni et al. 2004) compared with the software compat.py (Kauff and Lutzoni 2002) available at www.lutzonilab.net/downloads. A conflict was assumed to be significant if two different relationships for the same set of taxa (one being monophyletic and the other non-monophyletic) were observed in rival trees.

## RESULTS

### Phylogenetic analyses

The combined and ITS-only datasets comprise respectively sequences from 50 (49 ITS, 44 *ACT*, 43 *TUB2*, and 44 *RPB2*) and 108 collections including the outgroups. The best-fit loci selected by AIC were: GTR + I + G for ITS partition, K80 for 1st and 2nd codon position of *ACT,* GTR + G for the 3rd codon position of *ACT*, SYM + I for the for 1st and 2nd codon position of *TUB2*, GTR + G for the 3rd codon position of *TUB2*, GTR + I + G for the for 1st and 2nd codon position of *RPB2,* and HKY + I + G for the 3rd codon position, and finally GTR + I + G for the combined introns partition.

The final combined DNA sequence alignments of these loci, including gaps, resulted in 4368 characters (ITS: 1001 characters; *ACT*: 337 characters, of which 113 in the exon partition and 224 in the combined introns partition; *TUB2*: 1827 characters, of which 1049 in the exon partition and 778 in the combined introns partition; RPB2: 1203 characters, of which 1144 in the exon partition and 59 in the combined introns partition). No conflict involving significantly supported nodes was found between the tree topologies obtained for the individual datasets, using the 75% ML BS criterion; the datasets were therefore combined. The test of substitution saturation showed that the observed index of substitution saturation (Iss) for the *ACT*, *TUB2*, *RPB2,* the combined introns and ITS partitions, was significantly lower than the corresponding Iss.c, indicating that there was little saturation in our sequences (*P* < 0.001).

The two Bayesian runs converged to stable likelihood values after 520,000 generations. 7500 stationary trees (75% of total) from each analysis were used to compute a 50% majority rule consensus tree and to calculate posterior probabilities (BPP).

The consensus tree of the BI and the most likelihood tree of ML were congruent as far as the terminal clades or supported lineages are concerned thus only the Maximum Likelihood trees annotated with both BPP and ML BS are shown in Fig. 2 (combined dataset) and Fig. 3 (based on ITS sequences only).

The three specimens of *X. aurantiorubroguttata* cluster together in a highly supported clade (BPP = 1, ML BS = 100) phylogenetically close to a clade (BPP = 1, ML BS = 100) that comprises sequences of *Xylaria globosa* (Spreng. ex Fr.: Fr.) Mont. specimens (Fig. 2). The position of *X. aurantiorubroguttata* as sister to *X. globosa* results well-supported (BPP = 1, ML BS = 92). The clade encompassing *X. aurantiorubroguttata* and *Xylaria globosa* is sister to a clade formed by* X. spinulosa* Q.R. Li and J.C. Kang and *X. haemorrhoidalis*. In the ITS only phylogeny all collections of *Xylaria aurantiorubroguttata* cluster together in a well-supported terminal clade which also contains a *Xylaria* sp. specimen from Sri Lanka (Fig. 3, red box). So far, this clade remains isolated but is notably distantly related to all other *Xylaria* species for which sequences are known. Moreover, the ITS phylogenetic inference confirmed the position of *X. aurantiorubroguttata* as part of the *X. polymorpha* aggregate and resolved at least three clades identified as* X. scruposa* (Fr.) Fr., two clades identified as* X. atrosphaerica* (Cooke and Massee) Callan and J.D. Rogers and several species complexes such as* X. ophiopoda* Sacc./*X. schweinitzii* Berk. and M.A. Curtis species complex or the* X. enterogena* Mont. complex who arbour several phylogenetic species. On the other hand, all sequences available on GenBank (on 14/03/2024) and identified as* X. telfairii* (Berk.) Sacc., clustered out of the *X. polymorpha* aggregate and were excluded from the analysis.

Attempts to obtain ITS sequences via Sanger or NGS (Illumina) sequencing techniques from the type collections of *X. anisopleura* and *X. haemorrhoidalis* failed.

## TAXONOMY

**Fig. 4:**
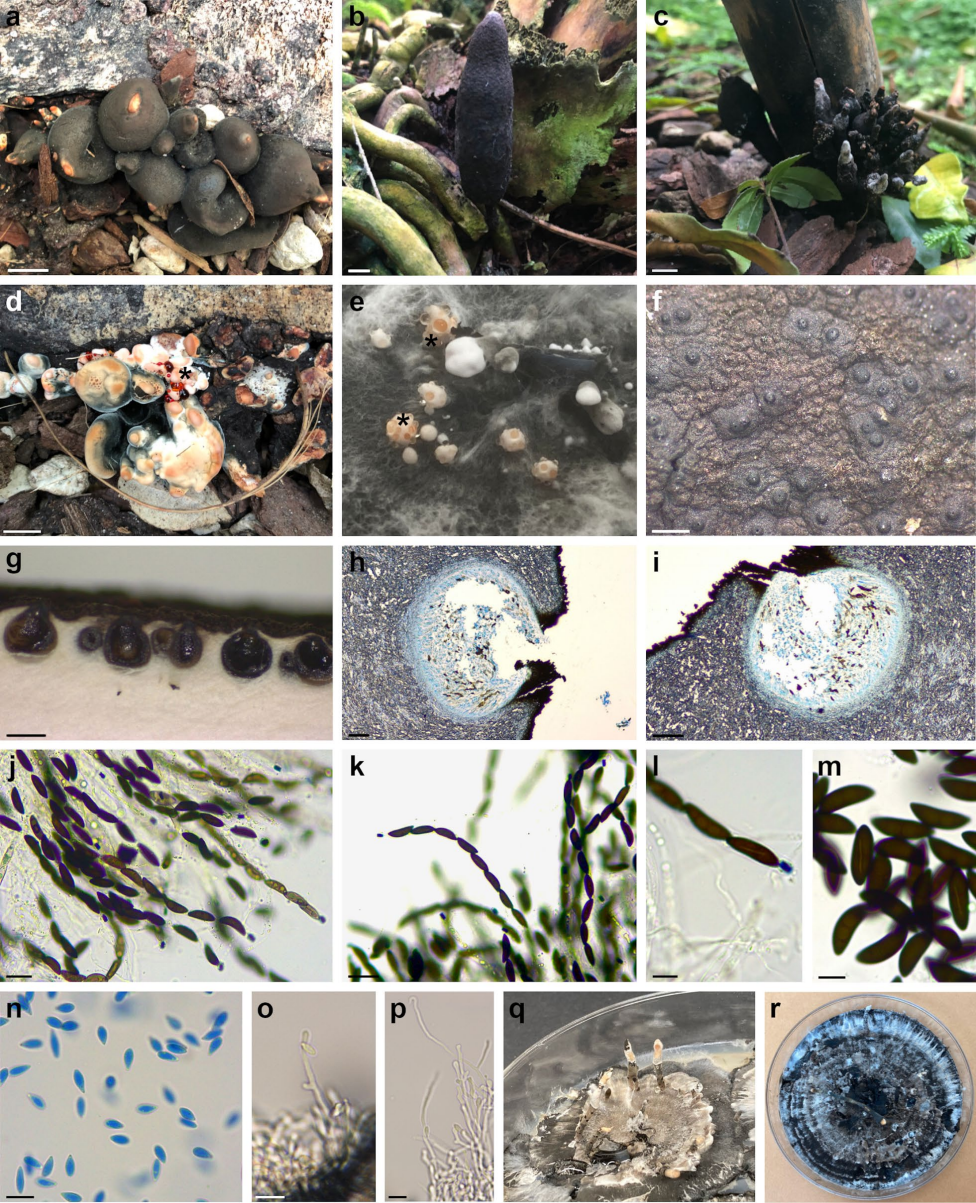
*Xylaria aurantiorubroguttata*. **a** Holotype PAD H0061559. **b** Paratype PAD H0061560. **c** Paratype PAD H0061561. **d** Immature stromata with red exudates (holotype, PAD H0061559). **e** Early stages growing on MEA with yellow-orange exudates. **f** Stromatal surface. **g**–**i** Horizontal section of ascoma. **j**, **k** Asci with ascospores and amyloid apical apparatus. **l**, **m** Ascospores showing germ slits. **n** Conidiospores found on the surface of immature stromata. **o**, **p** Conidiophores with conidiospores growing on MEA. **q** Colony on MEA in a 9 cm Petri dish at 6 weeks. **r** Colony on MEA in a 9 cm Petri dish at 12 weeks. Scale bars: **a**, **c** = 1 cm; **b** = 2 cm; **d** = 0.5 cm; **f**, **g** = 500 μm; **h**, **i** = 100 μm; **j** = 20 μm; **k** = 25 μm; **l**–**p** = 10 μm

**Fig. 5:**
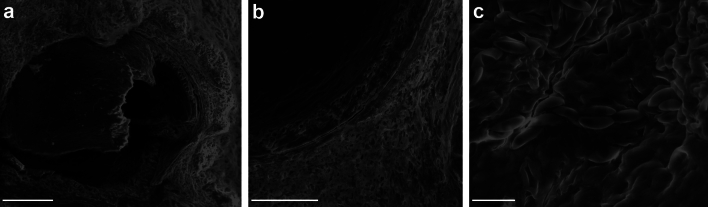
*Xylaria aurantiorubroguttata*. **a** Perithecium. **b** Longitudinal section of a perithecial wall. **c** Ascospores. Scale bars: **a** = 150 μm; **b** = 100 μm; **c** = 25 μm

***Xylaria aurantiorubroguttata*** N. Forin, Vizzini, M. Amalfi & S. Voyron **sp. nov.** Figures 4 and 5

MycoBank MB853488.

*Etymology*: The epithet was derived from the Latin words (adjectives) *aurantius* meaning “orange”, *ruber* meaning “red” and “*guttātus*” meaning spotted, covered with drops, and refers to the orange-red drops exudated by the immature stromata.

*Diagnosis*: Stromata upright, cylindrical, long-stipitate to globose to subglobose (penzigioid), short-stipitate; immature stromatal stages producing red (in nature) or yellow-orange (in Petri dishes) droplets; conidia obovoid (on average 8.8 × 3.8 μm) and ascospores ellipsoid-inequilateral, on average 23.9 × 8 μm, with a slightly oblique half spore-length non-sigmoid germ slit.

*Type*: **ITALY**: Padova, Botanical Garden. On *Delonix regia* stump, 11 November 2021, N. Forin (PAD H0061559—holotype preserved in the Padova Botanical Garden Herbarium; living culture TO MUT6703—ex-holotype stored in the Mycotheca Universitatis Taurinensis). **ITALY**: Padova, Botanical Garden. On *Phoenix reclinata* stump, 13 December 2021, N. Forin (PAD H0061560—paratype preserved in the Padova Botanical Garden Herbarium). **ITALY**: Padova, Botanical Garden. At the base of a bamboo rod, 13 December 2021, N. Forin (PAD H0061561—paratype preserved in the Padova Botanical Garden Herbarium).

*Sexual stage*: *Stromata* i) upright, solitary, fusiform to cylindrical, unbranched, with rounded apices, 7 cm total height, long-stipitate, fertile part 5 cm high × 1–1.5 cm wide, stipe 2 cm high × 0.2 cm wide, with a sterile rounded apex (Fig. 4b); ii) crowded in groups, short-stipitate, globose to subglobose, 1.8–3.2 cm total height, fertile part 1.5–2.5 cm high × 1–3 cm wide, stipe 0.3–0.7 cm high × 0.2–0.3 cm wide, sometimes with a pinched sterile apex (Fig. 4a). Young immature stromata, observed on *Delonix regia*, are characterized by a surface consisting of a white to orange outer layer producing orange to red exudates (Fig. 4d); mature stromata black, with an outer layer 30–40 μm thick, leathery, surface wrinkled, interior white and spongy consisting of thick-walled, 3.3–5.2 μm broad hyphae. *Perithecia* globose to subglobose, 500–550 μm high × 550–650 μm diam (Fig. 4g–i; Fig. 5a); perithecial wall about 40 μm (Fig. 5b). *Ostioles* dark black, papillate, 155–215 μm diam (Fig. 4f). *Asci* (6–)8-spored, cylindrical, long-stipitate, spore-bearing part 170–220 μm long × 8.7–10.4 μm wide, stipe 90–115 μm long, with an apical apparatus bluing in Lugol’s reagent, tubular to urn-shaped, 6.3–6.7 μm high × 3.5–3.8 μm wide (Fig. 4j–l). *Ascospores* (18.7–)22.4–*23.9*–25.3(–28.5) × (6.2–)7.4–*8*–8.7(–10.7) μm, Q = (2.1–)2.7–*3*–3.3(–4) (n = 250), brown, unicellular, uniseriate, ellipsoid-inequilateral, with slightly narrowly rounded or strongly pinched ends, smooth, mono or biguttulate, with a slightly oblique, straight half spore-length germ slit (average germ slit length = 11.8 vs. average spore length = 23.3, n = 10) (Fig. 4j–m; Fig. 5c).

*Culture characteristics*: Colonies reaching the edge of a 9 cm Petri dish in 14 days, at first white and cottony, becoming black, zonate, with black and white concentric zones. Reverse black. Early stages producing yellow to orange exudates (Fig. 4e). Immature stromata cylindrical, un-branched, up to 1.5 cm long by 0.2 cm diameter, black, white to orange or sometimes grey at tip (Fig. 4q).

*Asexual stage*: *Conidiophores* upright, smooth, hyaline. *Conidia* found both in Petri and on the surface of immature stromata, hyaline, smooth, ellipsoid to obovoid, (7.5–)8.2–8.9–9.6(–10.7) × (3.2–)3.5–*3.8*–4.1(–4.5) μm (n = 55) (Fig. 4n–p).

*Habitat*: Saprobic, on bark of *Delonix regia* and *Phoenix reclinata* stumps, in tropical and sub-humid environments of the Padova Botanical Garden greenhouse (Fig. 1). The two environments are characterized by a temperature that fluctuates from 24 to 28 °C and a humidity level from 60 to 70% depending on the season. The origin of the plants results unknown.

**Fig. 6:**
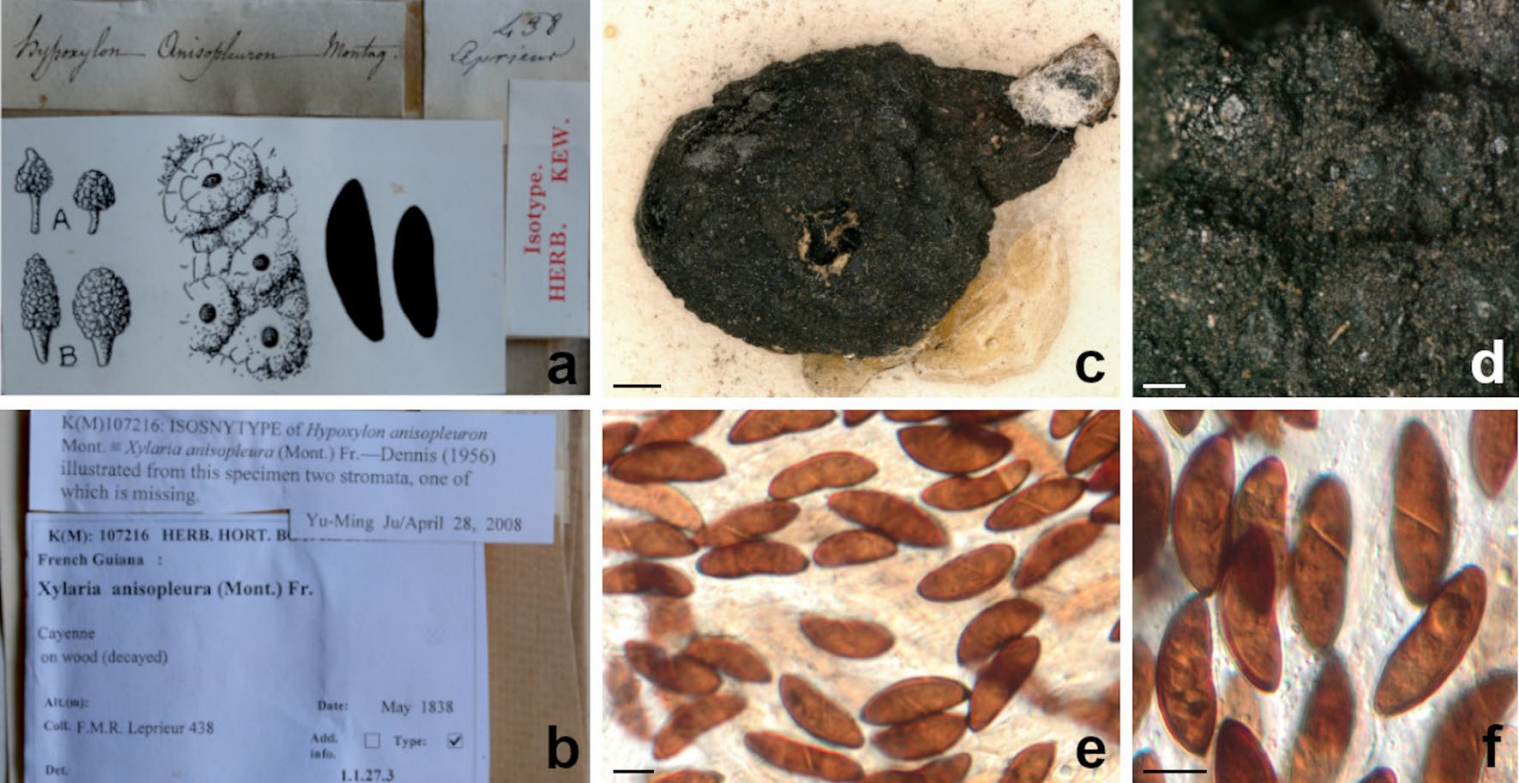
*Xylaria anisopleura*. **a**, **b** Original herbarium labels (K[M] 107,216, isosyntype of *Hypoxylon anisopleuron*). **b** Stromata. **c** Stromatal surface. **d** Ascospores. **e** Ascospores showing sigmoid germ slits. Scale bars: **b** = 1000 μm; **c** = 250 μm; **d**, **e** = 10 μm

***Xylaria anisopleura*** (Mont.) Fr., Nova Acta R. Soc. Scient. upsal., Ser. 3 1(1): 127 (1851) [1855]. Figure 6

*Basyonym: Hypoxylon anisopleuron* Mont., Annls Sci. Nat., Bot., sér. 2 13: 348 (1840).

*Type*: **FRENCH GUIANA**: Cayenne. On wood, May 1838, Leprieur, F. M. R. 438 (K[M] 107,216, – isosyntype of *Hypoxylon anisopleuron* Mont. preserved in the Royal Botanic Gardens Herbarium at Kew)*.*

*Sexual stage*: *Stromata* black, short-stipitate, subglobose, 0.79 cm high × 0.84 cm wide; stipe 0.27 cm high × 0.31 cm wide. *Perithecia* globose to subglobose, 570–700 μm diam. *Ostioles* dark black, slightly papillate. *Ascospores* (27.5–)25.8–*30.2*–34.6(–34.9) × (9.2–)8.9–*10*–11.1(–11.2) μm, Q = (2.5–)2.4–*3*–3.7(–3.8) (n = 20), brown, unicellular, ellipsoid-inequilateral, smooth, with a sigmoid germ slit.

**Fig. 7:**
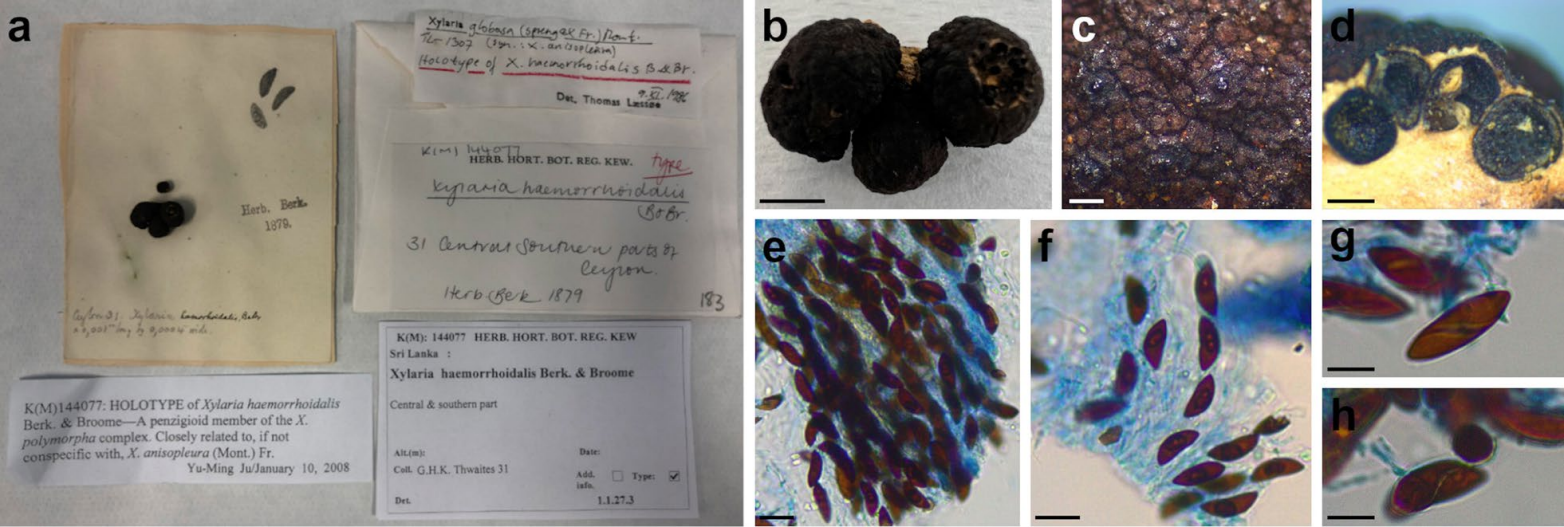
*Xylaria haemorrhoidalis*. **a** Original herbarium specimen (K[M] 144,077, holotype). **b** Stromata. **c** Stromatal surface. **d** Horizontal section of ascoma. **e**, **f** Ascospores. **g**, **h** Ascospores showing sigmoid germ slits. Scale bars: **b** = 5 mm; **c** = 350 μm; **d** = 500 μm; **e**–**g** = 20 μm; **h** = 10 μm

***Xylaria haemorrhoidalis*** Berk. & Broome, Journal of the Linnean Society, Botany 14: 117 (1875). Figure 7

*Type*: **SRI LANKA**: Central and southern parts. On wood, Thwaites, G.H.K. 31 (K[M] 144,077 – holotype preserved in the Royal Botanic Gardens Herbarium at Kew).

*Sexual stage*: *Stromata* penzigioid, aggregated, black, 0.6–0.8 cm high × 0.8–1 cm wide. *Perithecia* globose to subglobose, 500–1000 μm diam. *Ostioles* dark black, papillate, 125–175 μm diam. *Ascospores* (23.1–)24.9–*26.7*–28.5(–30.8) × (8.2–)9–*9.7*–10.4(–12.4) μm, Q = (2.3–)2.5–*2.8*–3(–3.8) (n = 50), brown, unicellular, ellipsoid-inequilateral, smooth, with a sigmoid germ slit.

## DISCUSSION

According to our multigene phylogenetic analysis,* Xylaria aurantiorubroguttata* clusters within the *X. polymorpha* aggregate (Fig. 2). The deeper phylogenetic analysis focusing only on the *X. polymorpha* aggregate, comprising all ITS sequences belonging to this group and available on GenBank, confirmed that indeed this is an extremely difficult group with many poorly circumscribed species often forming species complex. The ITS only tree showed at least three clades identified as *X. scruposa*, two clades identified as *X. atrosphaerica* and some complex of species such as *X. ophiopoda/X. schweinitzii* species complex or the *X. enterogena* complex which harbour several phylogenetic species. Moreover many “names” pasted on sequences appear scattered all over the phylogeny, this is the case of collections identified as *X. anisopleura* spanning in at least five different distantly related clades. Despite the clearly complex taxonomic situation, collections of *X. aurantiorubroguttata* remain isolated from all others in a highly supported clade. Only one unidentified *Xylaria* collection originating from Sri Lanka joins this clade, suggesting a Southeast Asian origin for the species, even though the plant species on which our *Xylaria* stromata grew have a Madagascar (*Delonix regia*, Babineau and Bruneau 2017) or South African distribution (*Phoenix reclinata* and *Strophanthus speciosus*, Van Wyk et al. 2005; Mjoli and Shackleton 2015), and the origin of the specimens cultivated in Padova is unknown (personal comm.).

Species within the *X. polymorpha* aggregate form a monophyletic group with high supports (BPP = 1, ML BS = 91) (Fig. 2) as already highlighted previously by other authors (e.g., Hsieh et al. 2010; Li et al. 2017; Konta et al. 2020) and are characterized by a wrinkled to minutely cracked stromatal surface and mostly 17–35 μm long ascospores usually bearing a less than spore length germ slit (Rogers and Callan 1986; Ju et al. 2009; Hsieh et al. 2010; Li et al. 2017).

*Xylaria aurantiorubroguttata* is distinguished by the presence of both upright, cylindrical, long-stipitate and globose to subglobose (penzigioid), short-stipitate stromata, immature stromatal stages producing in nature red drops while in Petri dishes yellow-orange ones, obovoid conidia (on average 8.8 × 3.8 μm), and ellipsoid-inequilateral ascospores, on average 23.9 × 8 μm, with a slightly oblique half spore-length non-sigmoid germ slit.

Based on our phylogenies the closest taxa to the new species are *X. haemorrhoidalis* and *X. globosa* (Figs. 2, 3).

*X. globosa*, a pantropical species (e.g., Rogers 1983; Rogers et al. 1988; San Martín and Rogers 1989; Van Der Gucht 1995; Ju and Rogers 1999; Hladki and Romero 2010; Fournier et al. 2018) which shares with *X*. *aurantiorubroguttata* the presence of highly variable in shape stromata, ranging from subglobose to clavate, ellipsoid, turbinate or fusiform, subsessile to long-stipitate, immature stromata covered with bright orange exudation droplets forming a thin orange pellicle upon drying, but it differs in a thicker stromatal crust (80–100 μm), bigger perithecia (600–900 μm diam), and longer ascospores, (20.6–)21.2–30.2(–31.4) × (6.3–)6.7–9.3(–10) μm (on average 25 × 7.9 μm), with strongly oblique to diagonal, slightly sigmoid, much less than spore-length germ slit (Fournier et al. 2018). Fournier et al. (2018) followed Van der Gucht (1995) in synonymizing *X. globosa* and *X. anisopleura* because the authors considered both species widely distributed and with high variable stromata morphology, and based on morphology the species cannot be unambiguously distinguished (Fournier et al. 2018). Our study cannot confirm this proposal as both species clustered together in two separate, distantly related terminal clades (Figs. 2, 3). Additionally, our morphological study of *X*. *anisopleura* isosyntype collection revealed bigger ascospores (on average 30.2 × 10 μm) than *X*. *globosa*. Thus, we considered that both names should be kept.

For what concerns *X. haemorrhoidalis*, synonymized with *X*. *allantoidea* (Berk.) Fr. by Dade (1940) and possibly with *X*. *anisopleura* by Dennis (1961) or *X*. *anisopleura* by Van der Gucht (1995), the species has been originally described from Ceylon (Sri Lanka) (Berkeley and Broome 1873) and then putatively reported also from Taiwan (Hsieh et al. 2010), Micronesia (strain SFC20160126-25, GenBank: MZ621205, Yim YW and Park MS, direct submission) and India (Boonmee et al. 2021) on molecular basis. Apart from the short and uninformative protologue (Berkeley and Broome 1873), a full morphological description of a collection determined as *X. haemorrhoidalis* is only reported in Boonmee et al. (2021) where, based on Indian specimens, the authors described it as a species with upright and long-stipitate stromata, ascospores (21–)23–26 × 10–12 μm (on average 25 × 10.5 μm), with a straight germ slit. However, the morphology of the holotype described here differs from that reported in Boonmee et al. (2021) especially in having penzigioid stromata and ascospores with a sigmoid germ slit (Fig. 6); this type of germ slit had already been highlighted for *X*. *haemorrhoidalis* by Ju et al. (2009). Consequently, based on morphological and/or molecular data, the Indian collection of *X*. *haemorrhoidalis* and *Xylaria* sp. from Thailand (Fig. 3) possibly represent a different species from *X. haemorrhoidalis* which is presumably represented by the clade containing the remaining sequences. Small penzigioid stromata, longer and wider ascospores with an evident sigmoid germ slit distinguish *X*. *haemorrhoidalis* from *X. aurantiorubroguttata*.

*Xylaria spinulosa* from southern China on deadwood of unknown plant, differs in having clavate stromata covered with long soft thorns, smaller ascospores 19–23 × 6.5–8.5 μm (on average 21.3 × 7.6 μm), with oblique to sigmoid germ slit, and no production of orange pigments and conidia on young stromata (Li et al. 2017).

*Xylaria schweinitzii* , one of the most common *Xylaria* species in the tropics (e.g., Rogers and Callan 1986; Rogers et al. 1988; San Martín and Rogers 1989; Van Der Gucht 1995; Ju and Rogers 1999; Carmona et al. 2009; Guzmán and Piepenbring 2011; Rogers and Ju 2012; Fournier et al. 2019), shares with *X*. *aurantiorubroguttata* the presence of amber-orange exudation droplets and obovoid to ellipsoid conidia, (7–)8–9 × 3–4(–4.5) μm on immature stromata both in natural and cultural conditions (Rogers and Callan 1986; Van der Gucht 1996), but is distinguished by a thicker stromatal crust, 70–80(–120) μm, bigger perithecia (600–850 μm diam), slightly smaller ascospores, on average 23 × 7.1 μm, with an oblique, occasionally slightly sigmoid germ slit 8–10 μm long, and yellowish extracellular granules at the base of the hymenium (Fournier et al. 2019).

The pantropical *X. scruposa* (Dennis 1956, 1958, 1961; Rogers and Callan 1986; Rogers et al. 1987, 1988; San Martín and Rogers 1989; Van Der Gucht 1995; Ju and Rogers 1999; Rogers and Ju 2012; Fournier et al. 2019) shows cylindric-fusiform to lanceolate stromata and smaller ascospores (14.2–)15.7–24.1(–28.1) × (4.9–)5.4–8.1(–9.1) μm (on average 19.4 × 6.5 μm), with a narrow, oblique, straight to most often slightly sigmoid germ slit ca. 1/2 spore-length (Fournier et al. 2019).

Finally, *X. polymorpha*, reference species for this species aggregate (complex), is a temperate taxon (Europe, North America) with cylindric to cylindric-clavate to spathulate large stromata, (2–)5–8(–15) × 0.5–2(–3) cm diam, large perithecia (500–1000 μm diam), no exudates on young stromata, and straight to slightly oblique ascospores with very long germ slit (up to 2/3 spore-length) (Breitenbach and Kränzlin 1981; Dennis 1981; Rogers and Callan 1986; Whalley 2000; Medardi 2006; Fournier 2014; Læssøe and Petersen 2019).

## CONCLUSIONS

In this study we presented a new *Xylaria* species characterized by a polymorphic stromata. Due to this characteristic, we are not surprised that the new species belongs to the *X. polymorpha* aggregate in the PO clade (Hsieh et al. 2010). Nevertheless, our phylogenetic analyses seem to indicate the existence of a complex taxonomic situation due to the possible non-correct morphology-based identification of many *Xylaria* specimens. The ITS phylogeny circumscribed to *Xylaria* species of the *X. polymorpha* aggregate (Fig. 3) showed the presence of different clades identified as the same species (e.g., *X. scruposa*, *X. atrosphaerica*), complexes of species (e.g., *X. ophiopoda*/*X. schweinitzii*) and specimens, identified as the same species, spanning in separate clades (*X. anisopleura*). The need to bring order within this genus is clear. However, most of the specimens used in the ITS-based phylogeny are represented in GenBank only by an ITS sequence, and it is now recognized that this molecular marker is not always informative enough as fungal barcode. Cedeño‐Sanchez et al. (2024) recently discovered a high level of intragenomic polymorphisms in the ITS region in *Hypoxylaceae* species, proposing the *TUB2* gene as a new primary barcode for *Hypoxylaceae* and other *Xylariales*. Given the limitations of the ITS region as barcode, we encourage the fungal taxonomists to deposit as much molecular information as possible in public databases, even when already described species are collected. Although molecular analysis of ancient type specimens can sometimes fail, it is still important to attempt their use for accurate and comprehensive taxonomic identification.

## Data Availability

ITS and combined alignments used for the phylogenetic analyses are deposited in Figshare (https://doi.org/10.6084/m9.figshare.25664241). The new generated sequences are deposited in GenBank as reported in the main text.

## Abbreviations

*ACT*: α-Actin
BPP: Bayesian posterior probability
CTAB: Cetyltrimethylammonium bromide
ESEM: Environmental scanning electron microscope
ITS: Internal transcribed spacer
MEA: Malt extract agar
ML BS: Maximum likelihood bootstrap support
MUT: Mycotheca Universitatis Taurinensis
PAD: Herbarium code of Padova botanical garden
*RPB2*: RNA polymerase subunit II
TO: Herbarium code of Torino botanical garden
*TUB2*: β-Tubulin
